# Supplementary material for: Mapping the CP-Transgene Insert in the Papaya Genome and Developing a Hermaphrodite Transgenic Hybrid with Broad-Spectrum Resistance to Papaya Ringspot Virus
Source: Viruses. 2024 May 22;16(6):823. doi: 10.3390/v16060823 (PMC11209241; doi:10.3390/v16060823)
Supplement: Supplementary file 1 [file viruses-16-00823-s001.zip › viruses-3007738-supplementary.pdf]

Supporting Information

Supplemental Table S1. Horticultural traits of transgenic and non-transgenic Tainung No.2 papaya fruits [a]

Fruit feature	Transgenic	Non-transgenic
Weight (gram per fruit)	1171.8 ± 121.3	1243.3 ± 162.2
Longitudinal length (cm)	23.9 ± 1.6	23.2 ± 1.0
Cross diameter (cm)	11.2 ± 0.4	11.1 ± 0.7
Sweetness (^{o}Brix)	12.6 ± 0.6	12.8 ± 0.8

[a] There is no difference of horticultural traits between transgenic and non-transgenic plants (unpaired Two-Samples T Test with p value >0.05, n=10.). The plants were grown under netting to avoid virus infection.
